# What Sex-Specific Differences Tells Us About Additive vs Synergistic Risk Prediction in Asthma

**DOI:** 10.1016/j.chpulm.2025.100134

**Published:** 2025-01-07

**Authors:** Samuel Mailhot-Larouche, Philippe Lachapelle, Simon Couillard

**Affiliations:** Faculté de médecine et des sciences de la santé, Université de Sherbrooke, Sherbrooke, QC, Canada

Risk prediction is an imprecise art, particularly evident in the field of asthma. An extensive effort by the Global Initiative for Asthma, an international panel of experts who review the literature, proposes a list of 20 risk factors.^1^ This list is based on 35 publications, none of which informed the multivariable prognostic relations across disease severities. This approach faces 3 major limitations.

First, we assess a given patient for each risk factor independently, adding up the positive prognostic characteristics to determine whether this person’s asthma is high risk and thus requiring treatment step-up.^1^ This purely additive approach means we ignore the overall value of a constellation of risk factors being present, that is we never consider that the whole (total risk) may be larger than the sum of the parts (total risk = baseline asthma attack rate × rate ratio of parameter A × rate ratio of parameter B × . . .). Clinicians know that the reality is more complex than this math.

Second, we evaluate many risk factors, not focusing on those that are mechanistically linked to the outcome which we strive to predict: asthma attacks. For instance, a history of asthma attacks or female sex increases risk but lacks a targetable mechanism. Other risk factors (eg, lung function, symptoms) are modifiable independently of an impact on asthma attacks. For example, long-acting bronchodilator monotherapy improves airflow and symptoms but shows inconsistent and limited effect on reducing asthma attack risk.^2^ Ideally, we would conjugate risk stratification with mechanistic targeting, focusing on prognostic factors that inform on a causal pathway (eg, type 2 inflammation),^3^, ^4^, ^5^ which is also targetable in a manner which removes the excess risk of asthma attacks (eg, with type 2-targeting antiinflammatory therapy).^6^^,^^7^

Third, we lack a well-validated, easily understood, and widely implemented risk stratification tool. In asthma, there are numerous prediction models that have been studied, but none have been validated nor adopted.^1^^,^^8^ The obvious example to follow is SCORE, a cardiovascular risk chart where the focus is on the impact of modifiable factors (eg, BP, cholesterol) alongside unmodifiable risk factors (eg, age, sex).^9^ How could we also harness multiple facets, embracing complexity with a simple tool?

In this issue of *Chest Pulmonary*, Agarwal et al^10^ shed new light on the value of integrating synergistic prognostic effects to better predict asthma attacks. The authors used a retrospective electronic health record-based cohort of 70,939 patients with asthma to predict the annualized rate of severe asthma attacks according to patient characteristics. This methodology is well established, with the added value of (1) integrating the blood eosinophil count and (2) exploring statistical interaction between blood eosinophils, female sex, and obesity. Blood eosinophil count is important because of the increasing literature establishing the blood eosinophil count not only as a predictive marker of therapeutic response,^4^^,^^6^ but also a strong prognostic marker of asthma attacks.^3^ Exploring statistical interaction is significant because a positive statistical interaction is viewed as a marker of synergy, whereby the combination of risk factors X and Y is associated with greater risk than either factor separately. Importantly, the authors use spline curves to illustrate nonlinear relationships and convert rate ratios into clinical insights. In effect, splines translate rate ratios to clinical numbers.

The authors share a table of unadjusted and adjusted rate ratios; however, the true value of this paper indeed resides in its spline curves (found in Figs 1 and 3).^10^

**Figure 1:**
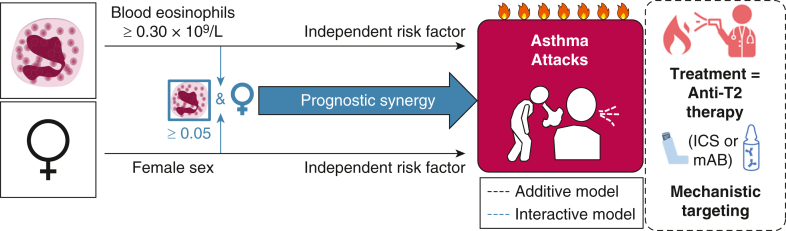
Additive vs integrative risk stratification. In conventional additive risk stratification, independent risk factors (eg, female sex, blood eosinophil count ≥ 0.3 × 10^9^/L) are used to determine the risk of asthma attacks. In keeping with the analyses by Agarwal et al,^10^ it appears that the purely additive view misses the important value of positive statistical interaction between the female sex and blood eosinophils, whereby female patients gain risk starting at a blood eosinophil count of 0.05 × 10^9^/L. We think this additional information gained by integrative risk stratification can help identify at-risk asthma benefiting from antiinflammatory treatment (eg, ICSs, mABs). ICS = inhaled corticosteroid; mAB = monoclonal antibody.

The first spline curve (Fig 1) confirms previous knowledge that the blood eosinophil count is a strong, independent, and dose-dependent risk factor for asthma attacks. This is important because the blood eosinophil count represents modifiable risk (ie, risk that is abrogated by appropriate antiinflammatory therapy).^6^

The second set of spline curves (Fig 3), conducted after observing 2 statistical interactions between (1) sex and blood eosinophils and (2) sex and obesity, brings forth novel additions to the field. Specifically, because Figure 3A’s curves disassociate between blood eosinophil counts of 0.05 to 0.3 × 10^9^/L, this suggest that female patients gain eosinophil-associated asthma attack risk at lower eosinophil counts than male patients, the latter only gaining risk at eosinophils ≥ 0.3 × 10^9^/L. This is particularly relevant because the threshold of 0.3 × 10^9^/L, considered significant in the field of asthma, could be inadequate for female patients (Fig 1). In Figure 3B, the authors investigated the combined value of obesity and female sex (which were found to positively interact), and in a rapid comparison of the panels, we conclude that female patients with obesity were at greater risk of asthma attacks, but the prognostic effect is milder than the contribution of eosinophilia.

To summarize, Agarwal et al’s^10^ most significant and interesting finding is that female patients gain eosinophil-attributable risk of asthma attack at blood eosinophil values of 0.05 to 0.3 × 10^9^/L, whereas male patients have a globally lower asthma attack rate which only increases with blood eosinophils ≥ 0.3 × 10^9^/L. Furthermore, obesity is an independent risk factor, most impactful in female patients of any type 2 phenotype. The main limitation of these data is the retrospective cohort design, which may affect the observed association between type 2 status and risk. This design does not assess the temporal relationship between active bronchial inflammation (type 2 status), which can vary over time, and subsequent exacerbations. We would be intrigued to evaluate the reproducibility of these interaction findings in prospective cohorts.

We view the interaction analysis by Agarwal et al^10^ as a significant advancement for ongoing risk prediction efforts in asthma.^11^ Notably, the clinical implication of statistical interactions between risk factors to predict asthma attacks is that the whole is indeed larger than the sum of the parts. Hence, to more precisely assess patients for future asthma attacks, we must devise a scale or a table that allows for the combination of risk factors to exponentiate the absolute risk.^3^^,^^11^ Only by assessing the patients comprehensively with clinical and inflammatory risk factors which target treatment opportunities (eg, obesity-driven risk implying the utility of weight loss, eosinophil-driven risk implying an opportunity for antiinflammatory therapy) can we improve the current management of asthma (Fig 1).^12^

## Funding/Support

Supported by the Association Pulmonaire du Québec’s Research Chair in Respiratory Medicine and the Fonds de Recherche du Québec.

## Financial/Nonfinancial Disclosures

The authors have reported to *CHEST Pulmonary* the following: S. M.-L. reports speaker honoraria from AstraZeneca, Sanofi-Regeneron, GlaxoSmithKline, Boehringer Ingelheim, and Novartis, outside of the submitted work; and received consultancy fees from AstraZeneca, GlaxoSmithKline and Sanofi-Regeneron, outside the submitted work. S. C. reports within the submitted work, he has received nonrestricted research grants from Sanofi-Genyme-Regeneron, bioMérieux, and the Québec Air-Intersectorialité-Respiratoire Network; and is the holder of the Association Pulmonaire du Québec’s Research Chair in Respiratory Medicine and is a clinical research scholar of the Fonds de recherche du Québec. Outside the submitted work, S. C. reports nonrestricted research grants from NIHR Oxford BRC, the Quebec Respiratory Health Research Network, the Fondation Québécoise en Santé Respiratoire, and AstraZeneca; having received speaker honoraria from AstraZeneca, GlaxoSmithKline, Sanofi-Regeneron, and Valeo Pharma; having received consultancy fees for FirstThought, AstraZeneca, GlaxoSmithKline, Sanofi-Regeneron, and Access Biotechnology and Access Industries; and having received sponsorship to attend/speak at international scientific meetings by/for AstraZeneca and Sanofi-Regeneron. S. C. is an advisory board member and holds stock options for Biometry Inc—a company which is developing a FeNO device (myBiometry), and has advised the Institut national d'excellence en santé et services sociaux (INESSS) for an update of the asthma general practice information booklet for general practitioners. None declared (P. L.).
